# Combination of IGF-1 gene manipulation and 5-AZA treatment promotes differentiation of mesenchymal stem cells into cardiomyocyte-like cells

**DOI:** 10.3892/mmr.2014.2812

**Published:** 2014-10-29

**Authors:** JUN LI, KAI ZHU, YULIN WANG, JIAYU ZHENG, CHANGFA GUO, HAO LAI, CHUNSHENG WANG

**Affiliations:** Department of Cardiac Surgery, Shanghai Institute of Cardiovascular Disease, Zhongshan Hospital, Fudan University, Shanghai 200032, P.R. China

**Keywords:** insulin-like growth factor-1, 5-azacytidine, mesenchymal stem cells, cardiomyocyte-like cells, differentiation

## Abstract

Mesenchymal stem cell (MSC) transplantation has been proposed as a promising therapeutic strategy for ischemic myocardium repair following myocardial infarction. Differentiation of MSCs into cardiomyocyte-like cells prior to cell transplantation is advantageous in improving their potential clinical benefits for cardiac repair. In the present study, we isolated and cultured porcine MSCs and evaluated the synergistic effect of 5-azacytidine (5-AZA) treatment and insulin-like growth factor-1 (IGF-1) gene manipulation on MSC differentiation into cardiomyocyte-like cells. Our results demonstrated that 5-AZA treatment alone induced a limited cardiomyocyte-like differentiation effect *in vitro*. Overexpression of the IGF-1 gene in MSCs improved the induction effect of 5-AZA, while knockdown of the IGF-1 gene attenuated the differentiation. These results suggest that IGF-1 is a significant stimulus affecting the cardiomyocyte-like differentiation of porcine MSCs. In addition, the combination of IGF-1 gene manipulation and 5-AZA treatment provides a new strategy to obtain more committed differentiated cardiomyocyte-like cells from porcine MSCs prior to cell transplantation.

## Introduction

Myocardial infarction (MI), often followed by progressive deterioration of left ventricular function, eventually leads to overt heart failure (1). In spite of recent therapeutic advances, heart failure following MI and left ventricular remodeling are still associated with significant morbidity and mortality rates. Mesenchymal stem cell (MSC)-based therapy is a promising treatment to prevent functional deterioration of MI towards heart failure. Following transplantation, MSCs differentiate into cardiomyocyte-like cells, which promote cardiac repair in the ischemic myocardium (2,3). However, the repair effect from cell therapy is modest or transient. A possible reason for this is the inefficient cardiomyocyte-like differentiation from transplanted MSCs *in vivo* (4,5). Emerging evidence suggests that differentiation induction of MSCs prior to transplantation improves the therapeutic effect from grafted cells (6–8). It has been shown that 5-azacytidine (5-AZA) induces the differentiation of MSCs into cardiomyocyte-like cells by random demethylation *in vitro*. However, the induction effect of 5-AZA alone is weak and limited (9,10). Therefore, the development of a more effective induction strategy is necessary for further research.

Insulin-like growth factor-1 (IGF-1), a 70-amino acid single-chain protein, plays a significant role in cardiomyocyte differentiation (11). Therefore, IGF-1 gene overexpression in MSCs may potentially improve the induction effect of 5-AZA for cardiomyocyte-like differentiation. In the present study, IGF-1 gene manipulation was combined with 5-AZA treatment to investigate the synergetic induction effect *in vitro*.

## Materials and methods

### Porcine MSC isolation and culture

Bone marrow was collected from the femur of five healthy female Shanghai white pigs (8–12 weeks old; Shanghai Jiagan Biological Technology Co., Ltd., Shanghai, China) with a 16-gauge needle containing 200 U/ml heparin (Sigma-Aldrich, St. Louis, MO, USA). The aspirates were depleted of mature blood lineages and purified by centrifugation in a 1.073-g/ml Percoll (Gibco, Grand Island, NY, USA) density gradient. Mononuclear cells were cultured in tissue culture flasks in low-glucose Dulbecco’s modified Eagle’s medium (LG-DMEM; Gibco) supplemented with 10% fetal bovine serum (Hyclone, Logan, UT, USA), 100 U/ml penicillin and 100 μg/ml streptomycin (Gibco). Non-adherent cells were discarded after 48 h, and adherent cells were further expanded until confluent. Cells were passaged up to eight times. The morphology of isolated cells was observed under a microscope (DM2700M; Leica, Wetzlar, Germany). This study was approved by the Institutional Review Board and Institutional Animal Care and Use Committee of Fudan University (Shanghai, China).

### Flow cytometry analysis

Cells were stained with CD90, CD44, CD34 and CD45 (Ebioscience, San Diego, CA, USA), and then with an appropriate secondary antibody conjugated with FITC and Cy5 (Ebioscience). After washing with phosphate-buffered saline (PBS), flow cytometry was performed on a BD FACSCalibur (BD Biosciences, San Jose, CA, USA).

### Cell proliferation assay

Cell proliferation was monitored by 3-(4, 5-dimethylthiazol-2-yl)-2, 5-diphenyltetrazolium bromide (MTT) assay. MSCs from passages 2, 5 and 8 were seeded onto 96-well plates (1×10^4^ cells/well), and cell proliferation was documented every 24 h for 12 days. The number of viable cells was assessed by measurement of the absorbance at 490 nm using a Safire 2 microplate reader (Tecan Systems Inc., San Jose, CA, USA).

### Cell vitality assay

MSCs from passages 2, 5 and 8 were prepared at a high concentration of 10^6^ cells/ml, and 10 μl cell suspension was mixed with 790 μl PBS and 200 μl trypan blue (20 g/l). The number of stained cells and the total number of cells were counted in a clean hemocytometer slide under a low-power microscope (DM2770M; Leica).

### Differentiation induction with 5-AZA alone

To demonstrate the single induction effect of 5-AZA (Sigma-Aldrich), MSCs from passages 2 and 5 were grown to 70–85% confluence and treated with 5-AZA (10 μmol/l) for 24 h. The morphological changes in MSCs from day 0 (prior to treatment) to day 21 were observed under a microscope (DM2700M; Leica).

### Construction of lentivirus encoding the IGF-1 gene or shRNA-IGF-1

cDNA encoding the IGF-1 gene was cloned and inserted into the pCDH-CMV-MCS-EF1-coGFP vector (GeneChem, Shanghai, China) at the *Eco*RI and *Xba*I sites. shRNA-IGF-1 was synthesized as follows: shRNA-IGF-1 sense, CCGGTGTTCAGG AAACAAG AACTACTCGAGTAGTTCTTGTTTCCTGAACTTTTTTG; and shRNA-IGF-1 antisense, AATTCA AAAAAGTTCAGGA AACAAGAACTACTCGAGTAGTTCTGTTTCCTGAACA. After annealing, the shRNA-IGF-1 fragment was cloned into the pLKO.1-TRC vector. The identities of recombinant plasmids pCDH-IGF-1-GFP and pLKO.1-shRNA-IGF-1 were confirmed by PCR and DNA sequencing.

Following construction, pCDH-IGF-1-GFP and pLKO.1-shRNA-IGF-1 were transfected into a packaging cell line (293T; American Type Culture Collection, Manassas, VA, USA) with package plasmids psPAX2 and pMD2.G (GeneChem) to produce a high-titer lentivirus. Concentrated viral supernatants were generated by ultracentrifugation and assessed by fluorescence-activated cell sorting (FACS) analysis for GFP on transduced control cells.

### Overexpression or knockdown of IGF-1 gene in MSCs

MSCs from passage 2 were divided into four groups as follows: i) MSC group: MSCs were untreated; ii) control group: MSCs were transfected with control lentivirus; iii) oeIGF-1 group: MSCs were transfected with lentivirus encoding IGF-1; and iv) siIGF-1 group: MSCs were transfected with lentivirus encoding shRNA-IGF-1.

### Follow-up treatment with 5-AZA in gene-manipulated MSCs

Following gene manipulation, MSCs from all groups (MSC, control, oeIGF-1 and siIGF-1) were grown to 70–85% confluence and then treated with 5-AZA (10 μmol/l) for 24 h. The dual-phase treated MSCs were then cultured for 21 days.

### Immunocytochemistry

One day after the combined induction, IGF-1 expression in all groups was verified by immunocytochemistry. Cells were fixed for 30 min with 4% formalin and rinsed with PBS. Following permeabilization with 0.4% Triton X-100 (Sigma-Aldrich) for 10 min, cells were blocked with 10% bovine serum albumin (Life Technologies, Grand Island, NY, USA) for 30 min prior to being incubated with goat anti-human polyclonal IGF-1 primary antibody (R&D, Santa Fe Springs, CA, USA) at 37°C for 2 h, followed by incubation in a 1:50 dilution of horseradish peroxidase-conjugated rabbit anti-goat IgG secondary antibody (R&D). Hematoxylin was used for nuclear counterstaining and the immunostained cells were visualized under a microscope (DM2700M; Leica).

### RNA isolation and quantitative polymerase chain reaction (qPCR)

On days 7, 14 and 21, IGF-1 expression and cardiomyocyte-specific markers (GATA-4, Nkx2.5, β-MHC and MEF2c) of the induced MSCs were identified by qPCR. Total RNA was isolated using TRIzol reagent (Invitrogen, Carlsbad, CA, USA) according to the manufacturer’s instructions. cDNA was prepared from 1 mg total RNA using a cDNA synthesis kit (Promega, Madison, WI, USA). qPCR was carried out with SYBR supermix (Takara, Shiga, Japan). The primers used for amplification are listed in Table I. The expression of each target mRNA relative to glyceraldehyde 3-phosphate dehydrogenase (GAPDH) was calculated based on the threshold cycle (CT) as r=2^−Δ(ΔCT)^.

### Immunoblotting

Cardiomyocyte-specific markers (GATA-4, Nkx2.5, β-MHC and MEF2c) of the induced MSCs were also identified with immunoblotting every week. Total protein was extracted from MSCs and differentiated cardiomyocyte-like cells, and then quantified with a BCA protein assay kit (Pierce, Rockford, IL, USA). Cell lysates were separated by SDS-PAGE (10%) and incubated with goat anti-pig polyclonal primary antibodies (anti-GAT4, -Nkx2.5, -β-MHC and -MEF2c; Santa Cruz Biotechnology, Inc., Santa Cruz, CA, USA) and donkey anti-goat IgG (R&D) secondary antibody conjugated with horseradish peroxidase. GAPDH was used as a loading control. Complexes were detected by chemiluminescence (Phototope-HRP Western Blot Detection System; Cell Signaling, Danvers, MA, USA).

### Statistical analysis

Each experiment was conducted at least three times. Data are presented as the means ± standard deviation. One-way analysis of variance was used to determine statistical significance between groups. Data were analyzed with SPSS version 17.0 software (SPSS, Inc., Chicago, IL, USA). P<0.05 was considered to indicate a statistically significant difference.

## Results

### Culture and characterizations of isolated porcine MSCs

**Figure 1:**
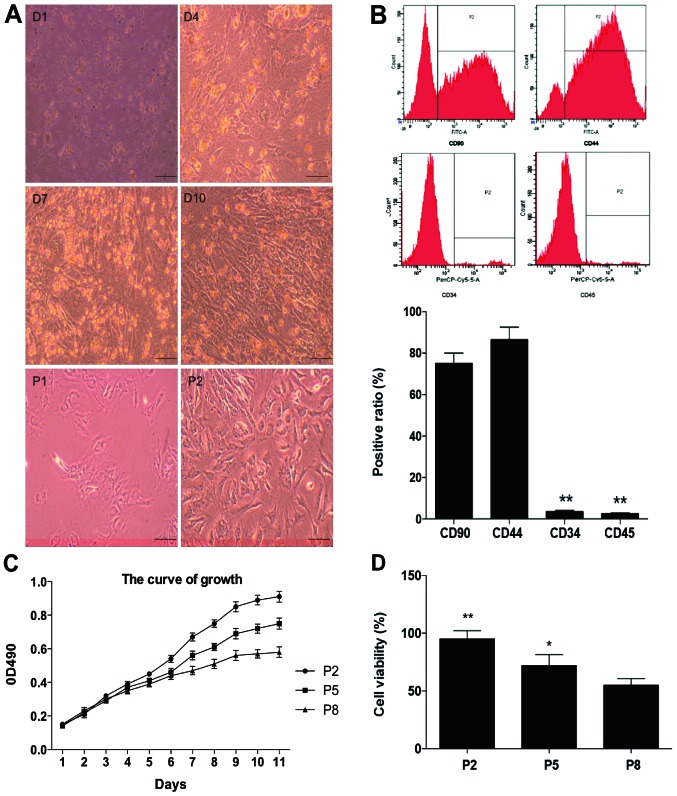
Morphology and characterization of isolated porcine mesenchymal stem cells (MSCs). (A) Morphology of MSCs at passage 2 (P2) on day 1 (D1), day 4 (D4), day 7 (D7) and day 10 (D10) show an adherent fibroblast-like shape and begin to form cell colonies after 10 days of culture. Passage 1 (P1) exhibited low growth ability. Scale bar, 50 μm. (B) Fluorescence-activated cell sorting analysis of CD90, CD44, CD34 and CD45 expression in MSCs. The histogram shows the average positive percentage of different antibodies from three independent experiments. Columns represent mean values and error bars represent SD. ^**^P<0.01, vs. positive antibody expression. (C) The proliferation rate of MSCs in different passages (P2, P5 and P8) was detected by proliferation assay. (D) The cell viability of MSCs in different passages (P2, P5 and P8) was detected by trypan blue staining. Columns represent mean values and error bars represent SD. ^*^P<0.05 and ^**^P<0.01, vs. P8.

The morphology of isolated porcine MSCs (passage 2) is shown in Fig. 1A. During the first two days, the culture was heterogeneous, containing round and non-adherent cells with lipid micelles in the supernatant. After 10 days, the cell population became more homogeneous, presenting an adherent fibroblast-like shape, and began to form cell colonies.

To characterize the MSCs, we performed flow cytometric analysis with antibodies against CD90, CD44, CD34 and CD45. As shown in Fig. 1B, the majority of cells expressed CD90 and CD44 at moderate to high levels, while these cells were negative for CD34 and CD45 (surface markers for hematopoietic stem cells). MSCs could be passaged up to eight times and the proliferation rate and cell viability of MSCs varied among the different passages, as shown in Fig. 1C and D. MSCs in the second passage grew faster and exhibited higher viability than MSCs in the fifth or eighth passage (P<0.05). The growth curve for each group showed a similar ‘S’ shape. The first two days represented the latent phase, and the logarithmic growth phase was from day 3 to 6. Days 7 and 8 represented the plateau phase.

### 5-AZA promotes the commitment of MSCs to myocardial differentiation

**Figure 2:**
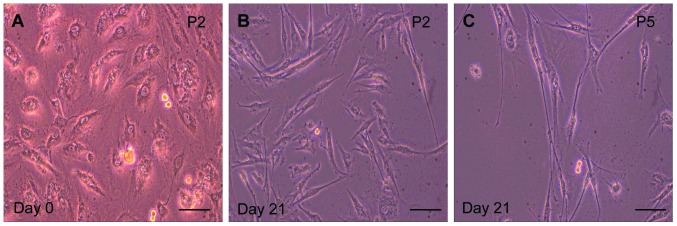
Morphological changes in mesenchymal stem cells (MSCs) treated with 5-azacytidine (5-AZA) for 21 days. (A) Morphology of porcine MSCs at passage 2 (P2) without 5-AZA treatment. (B) Morphology of P2 MSCs treated with 5-AZA for 21 days. (C) Morphology of passage 5 (P5) MSCs treated with 5-AZA for 21 days. Scale bars, 50 μm.

As shown in Fig. 2A, MSCs demonstrated a fibroblast-like morphology prior to 5-AZA treatment (0 weeks). Following 5-AZA treatment for 48 h, ~60% of the MSCs detached from the plate, and the morphology of the remaining cells gradually changed. The remaining 40% of the MSCs gradually increased in size, formed a ball-like appearance, or lengthened in one direction and formed a stick-like morphology at one week. As shown in Fig. 2B and C, the cells connected with adjoining cells after three weeks. MSCs in the second passage grew faster and exhibited a significantly higher cardiomyocyte-like induction ratio than those in the fifth passage (Table II).

### Overexpression of IGF-1 gene enhances myocardial differentiation of MSCs in the presence of 5-AZA

**Figure 3:**
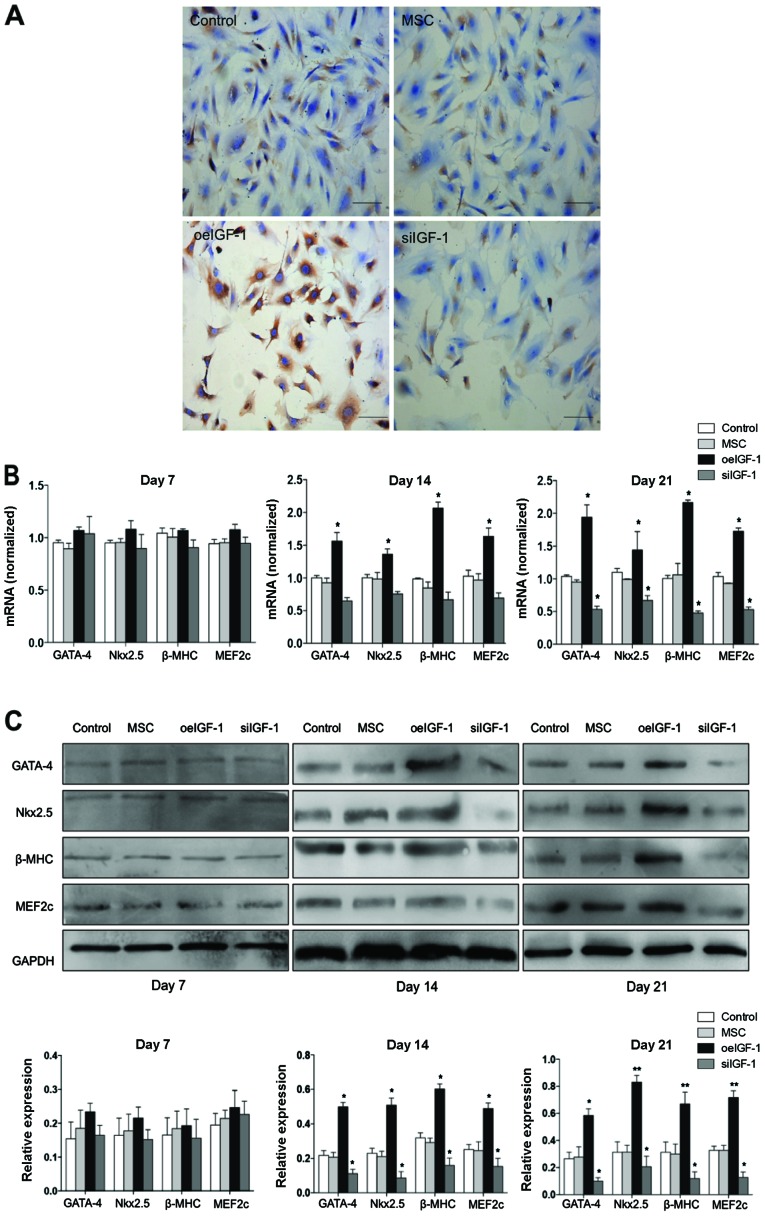
Insulin-like growth factor-1 (IGF-1) expression and cardiomyocyte-specific marker expression. (A) The expression of IGF-1 in different mesenchymal stem cell (MSC) groups was determined by immunocytochemistry. Scale bars, 50 μm. (B) Cardiomyocyte-like differentiation of MSCs with 5-azacytidine (5-AZA) treatment was detected by quantitative polymerase chain reaction, which was demonstrated by upregulation of GATA-4, Nkx2.5, β-MHC and MEF2c mRNA expression on days 14 and 21. Glyceraldehyde 3-phosphate dehydrogenase (GAPDH) was used as a reference gene. (C) Cardiomyocyte-like differentiation of MSCs with 5-AZA treatment was detected by western blot analysis, which was demonstrated by upregulation of GATA-4, Nkx2.5, β-MHC and MEF2c protein expression on days 14 and 21. Glyceraldehyde 3-phosphate dehydrogenase (GAPDH) was used as a loading control. MSC, untreated MSCs; control, MSCs transfected with control lentivirus; oeIGF-1 group, MSCs transfected with lentivirus encoding IGF-1; siIGF-1 group, MSCs transfected with lentivirus encoding shRNA-IGF-1. ^*^P<0.05 and ^**^P<0.01, vs. the control group.

IGF-1 expression in the oeIGF-1 group is shown in Fig. 3A. The combination of IGF-1 overexpression with 5-AZA treatment induced the cardiomyocyte-like differentiation of MSCs, which was demonstrated by the expression of specific cardiomyocyte markers (GATA-4, Nkx2.5, β-MHC and MEF2c). Compared with the MSC and control groups, the oeIGF-1 group expressed mRNA and protein of specific markers at a higher level on day 14 and day 21 following treatment with 5-AZA (P<0.05), but no significant difference was shown on day 7 (Fig. 3B and C).

### Knockdown of IGF-1 gene attenuates myocardial differentiation of MSCs

IGF-1 expression in the siIGF-1 group is shown in Fig. 3A. The mRNA and protein expression of the siIGF-1 group was significantly downregulated for the majority of the specific cardiomyocyte markers (P<0.05) (Fig. 3B and C).

## Discussion

MSCs differentiate into cardiomyocyte-like cells *in vivo* and promote cardiac repair in the ischemic myocardium. Pre-induction of the MSCs prior to transplantation improves the therapeutic effect significantly. In this study, a novel combined induction strategy, including IGF-1 gene manipulation and 5-AZA treatment, was developed for MSC differentiation into cardiomyocyte-like cells *in vitro*.

Until now, the mechanisms underlying the differentiation of MSCs into cardiomyocyte-like cells have been unclear. Numerous researchers believe that the paracrine effects of the cells are more likely to play a key role than any direct effect of the cells. A number of induction agents have been used to mimic the paracrine effect (12–15). 5-AZA, a DNA-demethylating chemical compound, induces the demethylation of CpG islands that normally remain unmethylated in the germ line, leading to an altered expression of certain genes that may regulate differentiation (16,17). Our study, consistent with previous literature, demonstrated that 5-AZA could induce a cardiomyocyte-like morphological change in MSCs. Furthermore, MSCs in the second passage exhibited better differentiation than those in the fifth passage when using a stand-alone treatment of 5-AZA, which indicated that the number of cell passages may be an influencing factor in the differentiation induction.

It has been demonstrated that IGF-1 promotes growth, proliferation and differentiation of numerous cell types, including cardiomyocytes and vascular smooth muscle cells, *in vivo* and *in vitro*, and inhibits cell apoptosis and necrosis. In addition, patients or experimental animals with IGF-1 deficiency show evidence of cardiac atrophy and impaired cardiac function (18–20). In the present study, the siIGF-1 group exhibited poorer differentiation than the control group, which indicates that the absence of the IGF-1 gene in MSCs inhibited their capacity of differentiation. Therefore, we hypothesize that intrinsic activation of the IGF-1 gene is essential to initiate the 5-AZA-based cardiomyocyte-like induction process.

IGF-1 treatment alone is not capable of inducing the differentiation of MSCs into cardiomyocyte-like cells, while the combination of multiple factors, including IGF-1, VEGF and bFGF, induces MSCs to differentiate and express cardiomyocyte markers under physiological conditions (21,22). These studies indicate that multiple factors are required during the differentiation process. The present study demonstrated that the combination of IGF-1 gene overexpression and 5-AZA treatment enhanced myocardial differentiation of MSCs for 21 days, which suggested a synergistic effect of IGF-1 gene manipulation and 5-AZA. It is known that IGF-1 binds to its receptor (IGF-1R), which possesses intrinsic tyrosine kinase activity and activates a number of downstream mediators, including phosphatidyl-inositol 3-kinase/Akt and MAP kinase (23,24). However, the exact molecular mechanism of IGF-1 gene manipulation involved in the cardiomyocyte-like differentiation of MSCs needs to be further explored in the future.

In conclusion, our findings demonstrate that the IGF-1 gene in MSCs is essential for cardiomyocyte-like differentiation. In addition, the combination of IGF-1 gene manipulation and 5-AZA treatment is feasible and effective for differentiation induction *in vitro*, which could be of significance for MSC-based cardiac repair in the ischemic myocardium.

## Figures and Tables

**Table I:** Target primers and product length.

Gene	Primer sequence (5′-3′)	Product length (bp)	Tm (°C)
IGF-1	TTCTACTTGGCCCTGTGCTTG	201	60.60
	CACACGAACTGAAGAGCGTC		59.22
GATA-4	CCTCCGGGGCCCTATGA	108	60.80
	GAGCACAGAGGAGGGCAC		61.50
MEF2c	CACCCTGCTGCTTTTACTATCCT	166	59.70
	GCTCAGCCCATTGTGACATTT		58.80
NKX2.5	GGGAGGAAGCGGCGAAC	156	61.50
	CGGTTGCCTGCTGACACG		60.80
β-MHC	CTGGGGCTCAAATGGTATGG	128	61.50
	GGCTGGTATTCAAAGGACGG		61.90
GAPDH	CTGGGCTACACTGAGCACC	101	62.00
	AAGTGGTCGTTGAGGGCAATG		62.90

Insulin-like growth factor-1; GAPDH, glyceraldehyde 3-phosphate dehydrogenase.

**Table II:** Cardiomyocyte induction ratio.

Passage number	Concentration of 5-AZA (μM)	General average of total cellular score (n)	General average of cardiomyocyte score (n)	Inductivity
P2	10	74.8	25.1	33.6^a^
P5	10	60.3	10.9	18.1^b^

Different letters in the same column indicate a significant difference (P<0.05). 5-AZA, 5-azacytidine.
